# Retract and Replace: The RasGAP-associated endoribonuclease G3BP assembles stress granules

**DOI:** 10.1083/jcb.20021212808022023r

**Published:** 2023-09-06

**Authors:** Hélène Tourrière, Karim Chebli, Latifa Zekri, Brice Courselaud, Jean Marie Blanchard, Edouard Bertrand, Jamal Tazi

Vol. 160, No. 6 | https://doi.org/10.1083/jcb.200212128 | March 17, 2003

The *Journal of Cell Biology* retracts Tourrière et al. (2003. https://doi.org/10.1083/jcb.200212128) further to findings described in Panas et al. (2019) and Tourrière and Tazi (2019) showing that the G3BP1 phosphomimetic mutant construct (pEGFP-C1-G3BP1-S149E) also contains an aberrant S99P mutation, invalidating a subset of the findings in Figs. 4 B and 5 B that relied upon this construct.

Since the remaining data that do not rely on this flawed construct continue to support the conclusion that G3BP1 regulates stress granule assembly and that Ras signaling modulates this process, the article has been replaced, removing the relevant sections of Figs. 4 B and 5 B and the conclusions drawn from those experiments (2023. https://doi.org/10.1083/jcb.200212128072023new).

Authors Hélène Tourrière, Karim Chebli, Latifa Zekri, Edouard Bertrand, and Jamal Tazi agree with the retraction of Tourrière et al. (2003) and replacement with Tourrière et al. (2023) as described above. We were unable to contact authors Brice Courselaud and Jean Marie Blanchard.

## References

[bib1] Panas, M.D., N. Kedersha, T. Schulte, R.M. Branca, P. Ivanov, and P. Anderson. 2019. Phosphorylation of G3BP1-S149 does not influence stress granule assembly. J. Cell Biol. 218: 2425–2432. 10.1083/jcb.201801214 31171631 PMC6605800

[bib2] Tourrière, H., K. Chebli, L. Zekri, B. Courselaud, J.M. Blanchard, E. Bertrand, and J. Tazi. 2003. The RasGAP-associated endoribonuclease G3BP assembles stress granules. J. Cell Biol. 160: 823–831. 10.1083/jcb.200212128 12642610 PMC2173781

[bib3] Tourrière, H., and J. Tazi. 2019. Reply to “Phosphorylation of G3BP1-S149 does not influence stress granule assembly.” J. Cell Biol. 218: 2433–2434. 10.1083/jcb.201905105 31171633 PMC6605805

[bib4] Tourrière, H., K. Chebli, L. Zekri, B. Courselaud, J.M. Blanchard, E. Bertrand, and J. Tazi. 2023. The RasGAP-associated endoribonuclease G3BP mediates stress granule assembly. J. Cell Biol. 10.1083/jcb.200212128072023new PMC10482220 37672657

